# A Nomogram Predicting Microvascular Invasion Risk in BCLC 0/A Hepatocellular Carcinoma after Curative Resection

**DOI:** 10.1155/2019/9264137

**Published:** 2019-07-25

**Authors:** Shuai-Xiang Gao, Rui Liao, Hua-Qiang Wang, Dan Liu, Fang Luo

**Affiliations:** ^1^Department of Hepatobiliary Surgery, The First Affiliated Hospital of Chongqing Medical University, Chongqing 400016, China; ^2^Department of Hepatobiliary Surgery, The People's Hospital of Nanchuan, Chongqing 408400, China

## Abstract

**Background:**

Numerous studies have shown that hepatocellular carcinoma (HCC) without microvascular invasion (MVI) may have better outcomes. This study established a preoperative MVI risk nomogram mainly incorporating three related risk factors of MVI in BCLC 0/A HCC after surgery.

**Methods:**

Independent predictors for the risk of MVI were investigated, and an MVI risk nomogram was established based on 60 patients in the training group who underwent curative hepatectomy for BCLC 0/A HCC and validated using a dataset in the validation group.

**Results:**

Univariate analysis in the training group showed that hepatitis viral B (HBV) DNA (P=0.034), tumor size (P<0.001), CT value in the venous phase (P=0.039), CT value in the delayed phase (P=0.017), peritumoral enhancement (P=0.013), visible small blood vessels in the arterial phase (P=0.002), and distance from the tumor to the inferior vena cava (IVC) (DTI, P=0.004) were risk factors significantly associated with the presence of MVI. According to multivariate analysis, the independent predictive factors of MVI, including tumor size (P=0.002), CT value in the delayed phase (P=0.018), and peritumoral enhancement (P=0.057), were incorporated in the corresponding nomogram. The nomogram displayed an unadjusted C-index of 0.851 and a bootstrap-corrected C-index of 0.832. Calibration curves also showed good agreement on the presence of MVI. ROC curve analyses showed that the nomogram had a large AUC (0.851).

**Conclusions:**

The proposed nomogram consisting of tumor size, CT value in the delayed phase, and peritumoral enhancement was associated with MVI risk in BCLC 0/A HCC following curative hepatectomy.

## 1. Introduction

Hepatocellular carcinoma (HCC) was the second leading cause of cancer-related death worldwide in 2012 [1]. According to the HCC management guidelines from the EASL-EORTC, the recommended treatment modalities for early-stage HCC (Barcelona Clinic Liver Cancer- [BCLC-] 0/A) include hepatic resection, radiofrequency ablation, percutaneous ethanol injection, and liver transplantation [2]. Nevertheless, despite curative resection, the average tumor recurrence rate is as high as 50%-60% at 3 years after surgery, and the long-term survival is still unsatisfactory. Fortunately, some prognostic markers of HCC have been identified, which are closely associated with the outcomes of patients with HCC after hepatic resection, such as nodule number, tumor capsule, microvascular invasion (MVI), and preoperative serum aspartate aminotransferase (AST) and *α*-fetoprotein (AFP) levels [3–5]. Of note, MVI has been indicated to be one of the most robust predictors for early recurrence and overall survival following surgical resection or liver transplantation [3, 6, 7]. Accordingly, in an attempt to stratify expected survival outcomes and therapeutic selection, it is important to predict MVI risk in patients with early HCC.

For most cancers, vascular invasion or metastasis signifies systemic disease that is not curable with surgery. However, in real practice, preoperative MVI of HCC is clinically difficult to predict. Currently, the diagnosis of MVI is mainly based on postoperative histologic examination, which limits preoperative decision making and the identification of appropriate surgical procedures by surgeons. An increasing number of investigations have developed prognostic models using new imaging techniques other than biomarkers or various clinical indicators for the preoperative estimation of MVI risk in HCC patients [6, 8, 9]. For example, Lee et al. [10] found that magnetic resonance imaging (MRI) features were independent predictors of MVI in HCC. Moreover, quantitative features based on local binary patterns were reported to be useful for predicting MVI risk [11]. Thus far, there is still debate over which imaging modality best estimates preoperative MVI in HCC, especially in early-stage HCC after hepatic resection. The ability to select HCC patients who are at high risk of developing preoperative MVI not only facilitates prognostic prediction in patients with early HCC but also enables the identification of patients who might benefit from appropriate adjuvant therapies.

To the best of our knowledge, there have been few attempts to evaluate the diagnostic abilities of preoperative CT value in the delayed phase and peritumoral enhancement for predicting MVI in BCLC 0/A HCC. In this study, we aim to use preoperative CT value in the delayed phase and peritumoral enhancement to objectively identify predictors of MVI in resected BCLC 0/A HCC.

## 2. Patients and Methods

### 2.1. Patient Selection

A total of 89 patients with HCC admitted at Chongqing Medical University from January 2017 to June 2019 were retrospectively reviewed. Sixty and 29 patients were divided into the training and validation groups, respectively. Patient inclusion criteria are as follows: (1) HCC cases within the BCLC criteria 0/A, having undergone R0 tumor resection, with postoperative examination clearly showing hepatocellular carcinoma with MVI; (2) no preoperative treatment, such as transcatheter arterial chemoembolization; (3) preoperative imaging examination and operation interval shorter than one week, with no macrovascular tumor thrombus found. The exclusion criteria included incomplete laboratory or imaging data. General information, laboratory indicators, and imaging data from the enrolled patients were collected as shown in Table 1.

### 2.2. Image Analysis

**Figure 1:**
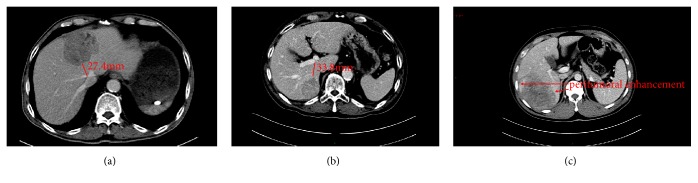
Typical CT images of patients with hepatocellular carcinoma.  Figure 1(a) shows the distance from the tumor to the IVC. The closest distance from the tumor to the inferior vena cava on the cross section of the venous-phase image was selected.  Figure 1(b) shows the distance from the tumor to the portal vein branches. When measuring the distance from the tumor to the portal vein branches, the portal vein branches were first positioned on the transverse section. Then, the shortest line from the portal vein branches to the tumor was drawn. As shown in Figure 1(c), we detected arterial-enhanced portions adjacent to the tumor border on arterial-phase images that became isodense with background liver parenchyma on delayed-phase images.

Patient CT images on the computer PACS system were independently examined by two investigators. Tumor size, tumor location, tumor capsule, tumor margin, peritumoral enhancement, the CT value at each phase, visible small blood vessels in the arterial phase, distance from the tumor to the IVC, distance from the tumor to the portal vein branches, and liver cirrhosis were assessed and measured. When measuring the tumor size, the longest diameter of the largest cross section was selected. When measuring CT values, the most obvious enhancement area of the largest cross section was selected, avoiding cystic changes, hemorrhage, and necrosis and circling the appropriate region of interest (ROI). Different periods were measured in the same plane. Peritumoral enhancement was defined as detectable arterial-enhanced portions adjacent to the tumor border on arterial-phase images that became isodense with background liver parenchyma on delayed-phase images [12, 13]. The closest distance from the tumor to the inferior vena cava on the cross section was selected. When measuring the distance from the tumor to the portal vein branches, portal vein branches were positioned on the transverse section (Figure 1).

### 2.3. Statistical Analysis

Statistical analyses were performed using SPSS software. Continuous variables were compared using the* t*-test or Mann-Whitney test. Categorical variables were compared using the **χ**2 test or Fisher exact test. A visual nomogram based on the results of multivariate logistic regression analysis was established using the rms package of R. The nomogram was obtained by proportionally converting each regression coefficient in multivariate logistic regression on a scale of 0 to 100 points. The total points represented the sum of points for each independent variable and were converted to predicted probabilities. The performance of the nomogram was measured by C-indexes and calibration with 1000 bootstrap samples to decrease overfit bias [14].

## 3. Results

### 3.1. Patient Characteristics

The baseline characteristics of the 89 patients in this study are shown in Table 1. In the training and validation groups, there were 50 and 26 men and 10 and 3 women, with a mean age of 51.7 and 57.8 years, respectively. In the training group and the validation group, the majority of patients had MVI (53.3% and 55.2%) and slightly higher mean levels of aspartate aminotransferase (ALT, 60.0 and 47.4 U/L, respectively) and gamma-glutamyl transpeptidase (GGT, 107.8 and 88.7 U/L, respectively) than normal (50 U/L). Most patients had large tumors (>3.0 cm, 6.0 cm, and 5.0 cm, respectively).

### 3.2. Univariate Analysis for Independent Predictors of MVI

As shown in Table 2, HBV DNA (*χ*2=4.499, P=0.034), tumor size (T=-3.940, P < 0.001), CT value in the venous phase (*χ*2=4.275, P=0.039), CT value in the delayed phase (*χ*2=5.720, P=0.017), peritumoral enhancement (*χ*2=6.104, P=0.013), visible small blood vessels in the arterial phase (*χ*2=9.902, P=0.002), and DTI (T=3.001, P=0.004) were significantly different between the two groups.

### 3.3. Multivariable Factors Associated with MVI

Logistic regression was employed to evaluate independent factors affecting MVI positivity. Tumor size (OR = 1.396, 95% CI 1.129-1.727; P = 0.002), CT value in the delayed phase (≤103.5 vs >103.5 OR=16.821, 95% CI 1.632-173.358; P=0.018), and peritumoral enhancement (absent vs present OR=5.220, 95% CI 0.955-28.542; P = 0.057) were independent predictors for MVI (Table 3). The probability of MVI positivity was calculated using the following formula:

(1) y^=11+exp.−xβ:y^=11+exp.2.756−0.334×tumor  size−2.823×CT  value  in  the  delay  phase  ≤103.5  vs>103.5,≤103.5=0,>103.5=1−1.653×peritumoral  enhancement  absent=0,present=1.

### 3.4. MVI Risk Prediction Nomogram

**Figure 2:**
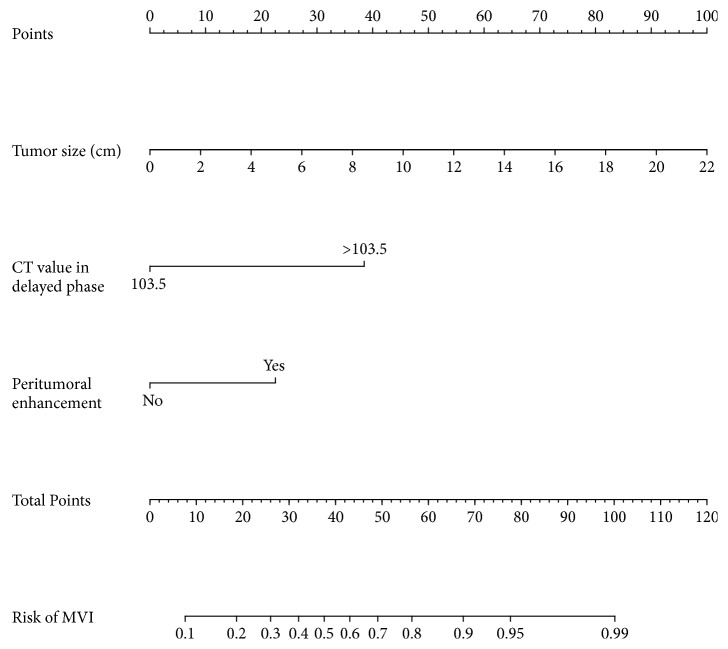
Nomogram to predict microvascular invasion (MVI) risk in BCLC 0/A hepatocellular carcinoma. To use the nomogram, find the score for each variable on the corresponding axis, add the points for all variables, and draw a line from the total points axis to the risk of MVI axis to determine the MVI risk.

**Figure 3:**
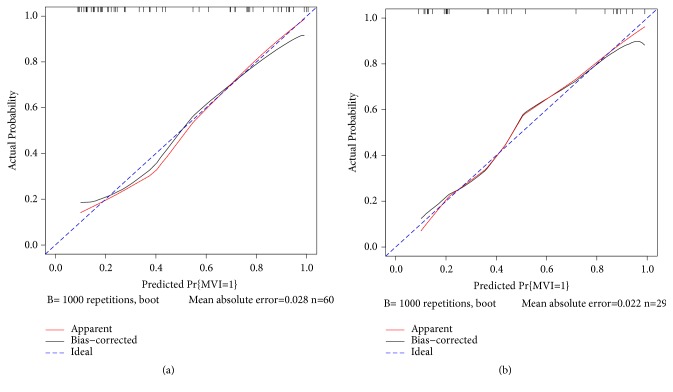
Calibration curves for the nomogram in estimating the risk of MVI in the training and validation groups. On the calibration curve, the x-axis is the nomogram-predicted probability of MVI, and the y-axis is the actual probability. The dotted blue line represents the ideal curve, the red line is the nomogram curve, and the black line is the bias-corrected curve.

To facilitate clinical use, we established a visual nomogram based on the results of multivariate logistic regression analysis using the rms package of R, as shown in Figure 2. In the training and validation groups, the nomogram displayed unadjusted C-indexes of 0.851 (95% CI, 0.761-0.951) and 0.861 (95% CI, 0.725-0.996) and bootstrap-corrected C-indexes of 0.832 and 0.862, respectively. Moreover, calibration curves showed good agreement regarding the presence of MVI between the nomogram prediction and histopathology diagnosis, as shown in Figure 3.

### 3.5. Selecting Optimal Cut-Off Values to Identify Patients with MVI

**Figure 4:**
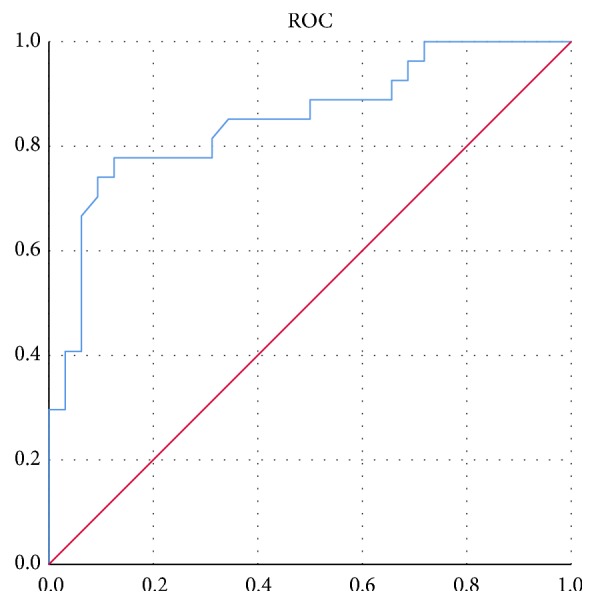
The ROC curve for the selected model. This curve was drawn with the total points for 60 patients in the training group. It sets a number of different critical values for total points to calculate a series of sensitivity and specificity values and then the curve is plotted with sensitivity as the ordinate and “1-specificity” as the abscissa. The larger the area under the curve, the higher the diagnostic accuracy. The AUC was 0.851.

An ROC curve was drawn with the total points for 60 patients in the training group (Figure 4). The area under the ROC curve for the selected model was 0.851 (95% CI 0.749-0.952, standard error 0.052), and the optimal cut-off value determined by maximizing Youden's index was 36.6818 ≈ 36.682 (sensitivity: 77.8%, specificity: 87.5%, Youden's index: 0.653).

## 4. Discussion

MVI is a microscopic feature that can significantly worsen the prognosis of early surgical HCC. However, preoperative diagnosis of MVI prior to treatment is almost impossible. The current study put forward three independent preoperative factors for predicting MVI in patients with BCLC 0/A HCC including tumor size, CT value in the delayed phase>103.5, and peritumoral enhancement. Based on these three predictors, we developed a new MVI nomogram combined with preoperative laboratory and imaging data.

In various studies, tumor size has been demonstrated to be an effective preoperative predictive factor for MVI [6, 9, 15]. It is well established that the prevalence of MVI in HCC is strongly influenced by tumor size [16, 17]. As the tumor grows, the risk of MVI continues to increase. In agreement with previous studies reporting the presence of MVI in 25% of tumors smaller than 2 to 3 cm [18], the current study revealed that a cut-off value of 3 cm could be used for MVI risk stratification.

In addition to clinical predictors of MVI, the identification of MVI from preoperative image analysis (e.g., ultrasound, CT, and MRI) has been attempted. In our study, peritumoral enhancement was a significant indicator of histologic MVI (P=0.013), in good agreement with the results of previous studies [10, 19, 20]. Some reports have suggested that the peritumoral enhancement area is where HCC microvascular invasion and satellite foci form along with the site of tumor venous drainage [12, 13, 21]. The venous drainage site gradually evolves into hepatic sinusoids and hepatic venules during the formation of cirrhotic nodules, atypical hyperplastic nodules, and early HCC. Hepatic sinusoids and venules in HCC merge with the surrounding parenchymal drainage veins. Therefore, in contrast-enhanced CT, the liver parenchyma around the tumor appears after tumor enhancement. Peritumoral enhancement usually appears in the late arterial or early portal venous phase and finally becomes isodense with the background liver parenchyma on delayed-phase images [20].

Here, for the first time, higher CT values in the delayed phase (>103.5) were found to be closely related to MVI in HCC. One explanation for continuous enhancement in the portal venous phase could be that the tumor is dually supplied by the hepatic artery and the portal vein. Microvascular invasion accelerates the release of tumor angiogenesis-promoting factors, such as hypoxia-inducible factor *α* (HIF1*α*) and vascular endothelial growth factor (VEGF), and diversifies the neovasculature supplying blood to tumors [22]. Tumor enhancement reflects the characteristics of the blood supply. This is likely because the tumor continues to release large amounts of HIF1*α* and VEGF and induces robust microangiogenesis. Moreover, delayed-phase enhancement is mainly related to the extravascular space and the permeability of blood vessels. Therefore, it is possible to predict microvascular invasion in HCC with prolonged enhancement.

In the current study, the C-index (0.851), calibration curve, and ROC curve analysis (0.851, 95% CI: 0.749-0.952) demonstrated that our nomogram was accurate in predicting MVI risk in surgical patients with BCLC 0/A HCC. However, this is a single-center retrospective study with a small sample size. The actual application efficiency of the scoring system may be affected. Therefore, multicenter and large-scale research is necessary to improve the scoring system. Moreover, a prospective study is required to further confirm the reliability of the nomogram.

In conclusion, based on three preoperative risk factors of MVI, we developed an objective scoring system to predict the MVI risk of HCC patients after curative resection and found an optimal cut-off point of 36.682. The model might help us make informed decisions based on expected survival outcomes and therapeutic assignment in patients with BCLC 0/A HCC. In the future, a large-scale prospective validation study is needed to assess the extensive applicability of the nomogram.

## Figures and Tables

**Table 1:** Patient characteristics.

Variable	Training group (n=60)	Validation group (n=29)	T/**χ**2	*P value*
Age, y	51.7±12.7	57.8±10.7	-2.238	0.028
Gender (male, female)	50/10	26/3	0.222	0.637
BMI, Kg/m^2^	22.7±3.7	23.2±2.5	-0.649	0.518
PLT,10^9^/L	195.9±142.6	138.1±79.3	2.031	0.045
ALB, g/L	40.7±6.0	40.0±4.2	0.660	0.511
TB, umol/L	18.0±22.1	13.6±14.9	0.957	0.341
ALT, U/L	60.0±77.1	47.4±43.4	0.816	0.417
AST, U/L	64.2±97.2	40.7±30.0	1.270	0.208
GGT, U/L	107.8±193.5	88.7±95.5	0.502	0.617
PT, S	13.7±1.0	13.7±1.0	-0.053	0.958
PTA, %	93.6±12.8	90.5±12.0	1.090	0.279
Liver Function Grading (A/B)	58/2	28/1	- - -	1.000
HBV DNA(<10e3, ≥ 10e3IU/mL)	46/14	19/10	1.234	0.267
AFP(≤400,>400ng/ml)	45/15	22/7	0.008	0.930
HCV (Absent, Present)	59/1	28/1	- - -	0.548
Tumor size, cm	6.0±3.9	5.0±3.7	1.092	0.278
CT value in unenhanced phase (≤42.5,>42.5)	28/32	6/23	5.588	0.018
CT value in artery phase(≤66.5,>66.5)	20/40	9/20	0.047	0.828
CT value in venous phase(≤102,>102)	44/16	20/9	0.185	0.667
CT value in delayed phase(≤103.5,>103.5)	51/9	24/5	0.002	0.970
Located in the left lobe (Absent, Present)	44/16	20/9	0.185	0.667
Capsule (Absent, Present)	44/16	25/4	1.860	0.173
With smooth margin (Absent, Present)	51/9	18/11	5.901	0.015
Peritumoral enhancement (Absent, Present)	47/13	24/5	0.237	0.626
Visible small blood vessel (Absent, Present)	21/39	15/14	2.270	0.132
The distance from the IVC	3.2±2.3	4.2±2.6	-1.540	0.127
The distance from the portal vein branches	3.6±2.3	5.0±2.5	-1.770	0.080
Liver cirrhosis (Absent, Present)	35/25	16/13	0.080	0.778
MVI (Absent, Present)	32/28	16/13	0.027	0.870

Abbreviations: BMI: body mass index; PLT: platelet; ALB: albumin; TB: total bilirubin; ALT: alanine aminotransferase; AST: aspartate aminotransferase; GGT: gamma-glutamyl transpeptidase; PT: prothrombin time; PTA: prothrombin activity; HBV: hepatitis B virus; AFP: alpha fetoprotein; HCV: hepatitis C virus; MVI: microvascular invasion.

**Table 2:** Univariate analysis of factors affecting MVI positivity in the training group.

Variable	MVI Negative	MVI Positive	T/**χ**2	*P value*
	(n=32)	(n=28)		
Age, y	53.9±12.9	49.1±12.1	1.489	0.142
Gender (male, female)	28/4	22/6	0.335	0.563
BMI, Kg/m^2^	22.7±3.9	22.8±3.5	-0.056	0.955
PLT,10^9^/L	165.1±99.9	231.1±174.9	-1.825	0.073
ALB, g/L	41.2±5.5	40.1±6.5	0.771	0.444
TB, umol/L	20.2±29.4	15.5±8.0	0.818	0.417
ALT, U/L	57.4±70.4	63.0±85.4	-0.278	0.782
AST, U/L	45.5±46.0	85.5±131.5	-1.615	0.112
GGT, U/L	102.6±242.6	113.8±119.0	-0.223	0.824
PT, S	13.8±1.2	13.4±0.8	1.503	0.138
PTA, %	91.8±14.1	95.6±11.1	-1.156	0.253
Liver Function Grading (A/B)	30/2	28/0	- - -	0.494
HBV DNA(<10e3, ≥ 10e3IU/mL)	28/4	18/10	4.499	*0.034*
AFP(≤400,>400ng/ml)	25/7	20/8	0.357	0.550
HCV (Absent, Present)	31/1	28/0	- - -	1.000
Tumor size, cm	4.3±2.7	7.9±4.1	-3.940	*<0.001*
CT value in unenhanced phase (≤42.5,>42.5)	17/15	11/17	1.149	0.284
CT value in artery phase(≤66.5,>66.5)	12/20	8/20	0.536	0.464
CT value in venous phase(≤102,>102)	27/5	17/11	4.275	*0.039*
CT value in delayed phase(≤103.5,>103.5)	31/1	20/8	5.720	*0.017*
Located in the left lobe (Absent, Present)	24/8	20/8	0.097	0.755
Capsule (Absent, Present)	24/8	20/8	0.097	0.755
With smooth margin (Absent, Present)	26/6	25/3	0.257	0.612
Peritumoral enhancement (Absent, Present)	29/3	18/10	6.104	*0.013*
Visible small blood vessel (Absent, Present)	17/15	4/24	9.902	*0.002*
The distance from the IVC	4.0±2.4	2.3±1.9	3.001	*0.004*
The distance from the portal vein branches	4.0±1.8	3.0±2.3	1.870	0.067
Liver cirrhosis (Absent, Present)	17/15	18/10	0.765	0.382

*∗* P < 0.05

Abbreviations: BMI: body mass index; PLT: platelet; ALB: albumin; TB: total bilirubin; ALT: alanine aminotransferase; AST: aspartate aminotransferase; GGT: gamma-glutamyl transpeptidase; PT: prothrombin time; PTA: prothrombin activity; HBV: hepatitis B virus; AFP: alpha fetoprotein; HCV: hepatitis C virus.

**Table 3:** Multivariate analysis of predictors for nomogram development.

Variables	*β*	standard error	OR	95%CI	P value
Tumor size	0.334	0.108	1.396	1.129-1.727	0.002
CT value in delayed phase	2.823	1.190	16.821	1.632-173.358	0.018
Peritumoral enhancement	1.653	0.867	5.220	0.955-28.542	0.057

Multivariate analysis: logistic regression model.

Abbreviations: OR: odds ratio; CI: confidence interval.

## Data Availability

The clinical data used to support the findings of this study were provided by Department of Hepatobiliary Surgery, the First Affiliated Hospital of Chongqing Medical University, and cannot be made freely available. Access to these data will be considered by the author upon request, with permission from the Director of the Department of Hepatobiliary Surgery of this hospital.

## Supplementary Materials

Supplementary Materials The file contains R codes.
