# Supplementary material for: A Nomogram Predicting Microvascular Invasion Risk in BCLC 0/A Hepatocellular Carcinoma after Curative Resection
Source: Biomed Res Int. 2019 Jul 25;2019:9264137. doi: 10.1155/2019/9264137 (PMC6683833; doi:10.1155/2019/9264137)
Supplement: Supplementary Materials — The file contains R codes. [file 9264137.f1.pdf]

```
# #Training   group
#nomogram
rm(list=ls())
library(survival)
library(survminer)
library(rms)
setwd("D:/R work")
infert <- read.csv("rawdata.csv")
infert=subset(infert,Group=="0")
infert <- data.frame(infert)
str(infert)                              # Check type of variables
summary(infert)                            # Statistical summary
attach(infert)
dd <- datadist(infert)
options(datadist="dd")

# Nomogram Plot
f<- lrm(MVI~TD+CT+PE       ,data=infert)
nom <- nomogram(f, fun=plogis,
                fun.at=c(.01, seq(.1,.9, by=.1), .95,.99),
                lp=F, funlabel="Risk of MVI")
plot(nom,xfrac=.25)
nom

# C-Index
f <- lrm(MVI~TD+CT+PE     , x=T, y=T,data = infert)
rcorrcens(infert$MVI ~ predict(f))
x = rcorr.cens(predict(f),MVI)
se   <-   x["S.D."]/2
Low95   <- x["C Index"] - 1.96*se
Upper95 <- x["C Index"] + 1.96*se
cbind( x["C Index"],      Low95, Upper95)

#####Harrell concordance index with Bootstrap Resampling Validation
library(rms)
set.seed(123)
dd<- datadist(infert)
options(datadist='dd')
```

```
f <- lrm(MVI~TD+CT+PE , x=T, y=T,data = infert)
# Get the Dxy
v <- validate(f, dxy=TRUE, B=1000)
Dxy = v[rownames(v)=='Dxy', colnames(v)=='index.corrected']
orig_Dxy = v[rownames(v)=='Dxy', colnames(v)=='index.orig']
# The c-statistic according to Dxy = 2(c-0.5)
bias_corrected_c_index <- abs(Dxy)/2+0.5
orig_c_index <- abs(orig_Dxy)/2+0.5
bias_corrected_c_index
orig_c_index
#####################################

#calbration curve
f <- lrm(MVI~TD+CT+PE, x=T, y=T,data = infert)
validate(f,method="boot",B=1000,dxy=T)
cal<-calibrate(f,method="boot",B=1000,predy= seq(0, 1, length=100),group=
MVI)
plot(cal,legend=FALSE)
lines(cal, lwd=1,lty=1,col="red")
abline(0,1,lwd=1,lty =2,col="blue")
legend("bottomright", cex=0.8, legend=c("Apparent", "Bias-corrected","Ideal"),
col=c( "red", "black", "blue"), lwd=c(1, 1, 1), lty=c( 1,1,2))

## Validation group
rm(list=ls())
library(survival)
library(survminer)
library(rms)
setwd("D:/R work")
infert <- read.csv("rawdata.csv")
infert=subset(infert,Group=="1")
infert <- data.frame(infert)
str(infert)                          # Check type of variables
summary(infert)                        # Statistical summary
attach(infert)
dd <- datadist(infert)
options(datadist="dd")
# C-Index
```

```
f <- lrm(MVI~score, x=T, y=T,data = infert) # Score was caculated on the basis of nomogram in training group.
rcorrcens(infert$MVI ~ predict(f))
x = rcorr.cens(predict(f),MVI)
se   <-  x["S.D."]/2
Low95   <- x["C Index"] - 1.96*se
Upper95 <- x["C Index"] + 1.96*se
cbind( x["C Index"],      Low95, Upper95)

#####Harrell concordance index with Bootstrap Resampling Validation
library(rms)
set.seed(666)
dd<- datadist(infert)
options(datadist='dd')
f <- lrm(MVI~score , x=T, y=T,data = infert)
# Get the Dxy
v <- validate(f, dxy=TRUE, B=1000)
Dxy = v[rownames(v)=='Dxy', colnames(v)=='index.corrected']
orig_Dxy = v[rownames(v)=='Dxy', colnames(v)=='index.orig']
# The c-statistic according to Dxy = 2(c-0.5)
bias_corrected_c_index <- abs(Dxy)/2+0.5
orig_c_index <- abs(orig_Dxy)/2+0.5
bias_corrected_c_index
orig_c_index
####################################

#calbration curve
f <- lrm(MVI~score, x=T, y=T,data = infert)
validate(f,method="boot",B=1000,dxy=T)
cal<-calibrate(f,method="boot",B=1000,predy= seq(0, 1, length=100),group= MVI)
plot(cal,legend=FALSE)
lines(cal, lwd=1,lty=1,col="red")
abline(0,1,lwd=1,lty =2,col="blue")
legend("bottomright", cex=0.8, legend=c("Apparent", "Bias-corrected","Ideal"), col=c( "red", "black", "blue"), lwd=c(1, 1, 1), lty=c( 1,1,2))
```
